# The Impact of Cell Phone Use on the Intensity and Liking of a Bout of Treadmill Exercise

**DOI:** 10.1371/journal.pone.0125029

**Published:** 2015-05-13

**Authors:** Michael J. Rebold, Andrew Lepp, Gabriel J. Sanders, Jacob E. Barkley

**Affiliations:** 1 College of Education, Health and Human Services, Kent State University, Kent, Ohio, 44242, United States of America; 2 Department of Exercise Science, Bloomsburg University of Pennsylvania, Bloomsburg, Pennsylvania, 17815, United States of America; 3 Department of Kinesiology and Health, Northern Kentucky University, Highland Heights, Kentucky, 41099, United States of America; National Research Council, ITALY

## Abstract

This study used a within-subjects design to assess the effect of three common cellular telephone (cell phone) functions (*texting*, *talking*, listening to *music*) on planned exercise. Forty-four young adults (*n* = 33 females, 21.8 ± 1.3 years) each participated in four, separate, 30-minute exercise conditions on a treadmill in a random order. During each condition, the treadmill speed display was covered and grade was fixed at zero. However, participants were able to alter treadmill speed as desired. Throughout the *texting* and *talking* conditions, research personnel used a pre-determined script to simulate cell phone conversations. During the *music* condition, participants used their cell phone to listen to music of their choice. Finally, participants completed a *control* condition with no cell phone access. For each condition, average treadmill speed, heart rate and liking (via visual analog scale) were assessed. Treadmill speed (3.4 ± 1.3 miles∙hour^-1^), heart rate (122.3 ± 24.3 beats∙min^-1^) and liking (7.5 ± 1.5 cm) in the *music* condition were significantly (*p* ≤ 0.014) greater than all other conditions. Treadmill speed in the *control* condition (3.1 ± 1.2 miles∙hour^-1^) was significantly (*p* = 0.04) greater than both *texting* and *talking* (2.8 ± 1.1 miles∙hour^-1^ each). Heart rate during the *control* condition (115.4 ± 22.8 beats∙min^-1^) was significantly (*p* = 0.04) greater than *texting* (109.9 ± 16.4 beats∙min^-1^) but not talking (112.6 ± 16.1 beats∙min^-1^). Finally, liking during the *talking* condition (5.4 ± 2.2 cm) was greater (*p* = 0.05) than the *control* (4.3 ± 2.2 cm) but not the *texting* (5.1 ± 2.2 cm) conditions. In conclusion, using a cell phone for listening to music can increase the intensity (speed and heart rate) and liking of a bout of treadmill exercise. However, other common cell phone uses (texting and talking) can interfere with treadmill exercise and reduce intensity.

## Introduction

According to the Pew Research Center (2013), 91% of American adults and 97% of young adults (age 18–29) own cellular telephones (cell phones). Indeed, the device is ubiquitous and its popularity is increasing along with its functionality. In addition to calling, most cell phones now provide many of the same services as an internet-connected computer. Currently, most modern cell phones allow users to access the internet, engage in diverse forms of social networking including text messaging, play video games, watch streaming video, and employ a wide array of specially designed software applications (apps). Because of the device’s inherent portability, users can now engage in all of these activities nearly anywhere and anytime. To illustrate, recent research has found that the cell phone is commonly used during work, academic pursuits such as classroom learning and studying, while watching movies and sporting events, during meals, and while going to the bathroom [1], [2]. In addition, Harrison and Gilmore’s study found the device is occasionally used in the shower, during religious services, on flights banning cell phone use, while having sex, and in other seemingly inopportune settings and circumstances. While the increased ability to connect to others and access information that cell phones provide likely has benefits, the concern here is that frequent cell phone use may become a distraction that in some circumstances negatively affects performance on other tasks.

An important daily task in which a distraction effect from cell phone use has been identified is driving an automobile. Research consistently shows that using a cell phone while driving is a distraction and significantly impairs performance [3–5]. One study famously compared the performance of cell phone drivers with intoxicated (i.e., drunk) drivers and found both to be similarly disadvantaged [6]. The decrease in performance measured in these studies is an example of the dual-task effect [7]. Simply put, when individuals simultaneously divide their attention between dual tasks, neither task receives the attentional resources it would have if it were attempted alone. Arguably, the importance of understanding cell phone related dual-task effects is growing as the use of these attention-demanding devices increasingly occurs during other activities [1], [2]. Accordingly, several studies have examined the dual-task effect of cell phone use on simulated street crossing behavior in a virtual environment with an integrated treadmill [7–9]. Findings consistently demonstrate that individuals using a cell phone for texting or talking take more time to initiate a street crossing and are more likely to error during a street crossing (e.g. disobey lights, cross into oncoming traffic) in comparison to an undistracted condition (i.e. no cell phone). This is true for older adults [7], college students [10], and early adolescents [11]. Cell phone related dual-task effects have been observed in naturalistic settings as well. Researchers have observed that pedestrians using cell phones for talking and texting, relative to undistracted pedestrians, are less likely to recall objects passed along the way even if those objects are extraordinary (e.g., a brightly colored clown on a unicycle) [12] and are more likely to display unsafe street crossing behaviors [13], [14].

In addition to distracting people during street crossings and other well-practiced pedestrian behaviors, cell phone use may also adversely affect the actual mechanics of walking. Parr, Hass, and Tillman recently utilized motion capture cameras to assess gait mechanics while walking and texting with a cell phone [15]. They noted that gait mechanics were adversely impacted and walking speed was reduced while texting and walking relative to a condition with no cell phone use. The combination of these impaired walking mechanics coupled with the distracting nature of cell phone use provides an explanation behind the greater risk of injury that has been linked with cell phone use while walking [16]. The strength of these studies is that most have utilized an experimental design, allowing for clear causal inferences to be made. Beyond this line of research, however, the use of experimental design to understand the effects of cell phone use on other common human behaviors is rare. For the most part, research in this area is exploratory and has focused on identifying relationships. For example, the relationship between cell phone use and academic performance is currently receiving much attention with most studies suggesting a significant negative relationship [17–20]. Recent research using a quasi-experimental design provides evidence that a distraction effect from cell phone use may be the cause of this negative relationship [21]. As more studies indicating relationships between cell phone use and various behaviors emerge, there is a need for studies able to assess the potential causal impact of these devices.

Recently our group began to explore the potential relationship between cell phone use and physical fitness [22]. With its increased functionality, the cell phone provides constant access to entertainment activities traditionally associated with increased sedentary behavior (e.g. surfing the internet, watching videos, playing video games) and sedentary behavior is negatively associated with physical activity and fitness [23–26]. Conversely, the cell phone is a mobile device and therefore can be used while standing, walking, and during some forms of moderate-intensity physical activity (e.g., pedaling a stationary exercise bike). Furthermore, an abundance of cell phone applications have been designed to increase physical activity and fitness. Thus, the relationship between cell phone use and physical activity/fitness may be different than the use of traditional sedentary devices (e.g., watching television). In our previous study, we assessed the relationship between cell phone use and cardiorespiratory fitness (i.e., peak oxygen consumption or VO_2_ peak ml kg^-1^ min^-1^) in a sample of typical college students. Results indicated that VO_2_ peak, which was assessed via indirect calorimetry during a progressive treadmill exercise test to exhaustion, was negatively associated with all measures of cell phone use (texting, calls made, and total minutes of use) [22]. This relationship was significant independent of several known correlates of VO_2_ peak: sex, percentage of body fat relative to lean mass, and self-efficacy for physical activity [23–26]. A better understanding of this relationship is important because poor cardiorespiratory fitness is a predictor for a number of health concerns [27], [28].

In an effort to explain this negative relationship between cell phone use and fitness, all participants were interviewed about their daily physical activities, sedentary behaviors, and cell phone use habits. Participants’ responses revealed two potential explanations for why cell phone use was negatively associated with cardiorespiratory fitness. First, participants tended to describe cell phone use as a sedentary behavior. Furthermore, high frequency cell phone users, relative to low frequency users, reported engaging in a greater number of sedentary behaviors (e.g. playing video games, watching TV, etc.). Therefore, cell phone use may be a marker for a wider array of sedentary behaviors which research suggests typically occurs at the expense of physical activity [23–26]. Second, participants identified cell phone use as a potential distraction during planned exercise and described how it may disrupt a given exercise bout. This idea is illustrated in the following statements from two different participants in response to the question: “Thinking about your daily life, would you say that your cell phone use increases or decreases your physical activity?” The participants replied:

*Maybe decrease*. *I’m not sure*. *I mean*, *sometimes I’ll be walking and texting on the treadmill*, *but it’s not like I’m stopping to send a text*, *I’m still walking*. *It is the same on the elliptical if I have to send a text*. *I’ve kind of gotten used to*, *like*, *balancing so that I can text if I need to*. *Or*, *if it’s too long*, *like with my mom*, *then I’ll call her and I’ll still be going on the treadmill*, *I might not be going as fast but I’m still going*.

*It decreases physical activity because*, *for instance*, *the other day*, *one of my friends called me during my work out*, *and like*, *I haven’t talked to her in a while and I had to tell her a lot of stuff*. *So it kind of distracted me from my work out*.

As these statements suggest, cell phone use during a bout of planned exercise may cause a distraction and potentially reduce exercise intensity. If so, this could limit the ability of an exercise bout to enhance fitness and provide a partial explanation for the negative relationship between cell phone use and cardiorespiratory fitness described above.

Thus, the purpose of this study was to assess the effect of cell phone use on a bout of 30-minutes of treadmill exercise. The study utilized a within-subjects design to compare the intensity (average speed and heart rate) and enjoyment (i.e., liking) of 30-minute bouts of self-selected treadmill exercise during the following conditions: texting on a cell phone (*texting*), talking on a cell phone (*talking*), using a cell phone to listen to music (*music*), and no cell phone use (*control*). It was hypothesized that using a cell phone to listen to music would result in the greatest average speed and liking of all conditions. This was hypothesized as listening to music has been previously shown to increase exercise intensity and enjoyment [29–33]. Conversely, we hypothesized that the other cell phone use conditions (*texting* and *talking*) would distract from treadmill exercise and result in decreased exercise intensity (i.e., reduced speed and heart rate) relative to the control condition. As such, this study is the first to utilize an experimental design to assess the effect of cell phone use on exercise behavior.

## Methods

### Participants

Forty-four college students (*n* = 33 females, *n* = 11 males, age 22.0 ± 1.4 years, Table 1) each participated in four separate 30-minute exercise conditions (*texting*, *talking*, *music*, and *control*) on a treadmill on separate days. The order of these conditions was randomized and each participant completed all four conditions (i.e., within-subjects design). Participants were excluded if they did not own a cell phone or had a cell phone without the ability to send text messages or replay music, and if they had any contraindications to exercise (i.e., orthopedic injuries). Prior to participation subjects were instructed on the benefits and risks of the study and signed informed consent and medical history forms. This study was approved by the Kent State University Institutional Review Board.

**Table 1:** Average height, weight, and age.

	Males (*n* = 11)	Females (*n* = 33)
Height (cm)	175.9 ± 9.8 cm	166.5 ± 8.0 cm
Weight (kg)	74.3 ± 14.5 kg	64.37 ± 12.59 kg
Age (years)	22.36 ± 1.75 years	21.58 ± 1.03 years

All data are means ± SD. No significant differences between males and females for height, weight, and age.

### Procedures

Participants reported to the Exercise Physiology Laboratory on four separate days. During each visit they completed one of the four exercise conditions (*texting*, *talking*, *music*, and *control*). This was a within-subjects design as each subject completed all four exercise conditions. Prior to initiation of each 30-minute exercise condition participants were familiarized with the treadmill (Quinton MedTrack CR60, Bothell, WA) and instructed that they would select the initial treadmill speed and were free to alter that speed at any time during the 30-minute session. In other words, if they wished to increase or decrease speed, at any point, participants were free to do so as often as they wished. It was necessary to allow participants to alter treadmill speed as assessing the effect of cell phone use on average speed was a primary purpose of this study. The treadmill grade was set at zero degrees and it was not to be altered. During each of the four exercise conditions time and speed displays on the treadmill were shielded from the participants using an opaque piece of paper. This was done in order to allow the participants to be able to alter their speed, but at the same time be blinded to the display.

Heart rate was measured every five minutes during all 30-minute exercise conditions (*texting*, *talking*, *music*, and *control*). Heart rate was recorded using a heart rate monitor (Polar, Kempele, Finland) and average heart rate was reported in beats min^-1^. Upon completion of each condition, liking of the condition was assessed by having participants place a mark on a 10 cm visual analog scale (VAS) anchored by “do not like it at all” on the left and “like it very much” on the right. Liking or hedonics is an affective rating of a behavior that directly correlates with physical activity participation. Average speed was calculated using the following formula: rate (mph) = distance (miles)/time (hours).

During the *talking* and *texting* conditions participants were interviewed over the phone (either via text message or conversation) by a member of the research staff. The interview consisted of the following seven topics: movies, songs, food, places to go on vacation, high school classes, hobbies, and places to go with friends on the weekend. For each of these topics the following questions were asked:

1. What is your favorite (insert topic here)?
2. How long has this been your favorite (insert topic here)?
3. When was the last time you (had, saw, heard, went to, participated in) your favorite (insert topic here)?
4. When do you hope to (have, see, hear, go to, participate in) your favorite (insert topic here) again?
5. Talk about the reasons why this is your favorite (insert topic here)?
6. If this is your favorite (insert topic here) what is your least favorite (insert topic here)?
7. Talk about the reasons why this is your least favorite (insert topic here)?
8. Tell me about the last time you (had, saw, heard, went to, participated in) your least favorite (insert topic here)?
9. If you could never (have, see, hear, go to, participate in) your favorite (insert topic here) again what would you do?
10. Would you like to add anything else regarding your favorite (insert topic here)?

The questions were repeated for each of the seven topics until the 30-minute treadmill condition ended. If a participant answered all 10 questions for each of the seven topics before the 30-minute treadmill condition concluded, then the research staff would repeat the questions for each topic until the 30-minute session expired. The research staff did not record the answers to these responses as these responses were considered inconsequential to the purpose of this study. The sole purpose of the interview was to simulate phone conservation (*texting* and *talking*). Research staff and participants were placed in separate rooms during the phone interview.

During the *music* condition participants used their cell phone to listen to music. Participants were instructed that they could listen to any kind of music that they desired. Participants were allowed to self-select their music as we believed this best simulated a real-world use of this cell phone function during exercise. Furthermore, we were concerned that a prescribed playlist of music would not be universally enjoyed by all participants and this lack of enjoyment could negatively affect exercise during that condition. Lastly, while there is evidence that the type (e.g., fast-paced versus slow-paced) of music listened to may differentially affect exercise, multiple studies have indicated that listening to music, regardless of type, has a positive effect on exercise behavior and/or perception [34–37].

During the *control* condition participants did not have access to their cell phone nor any interaction with other individuals or electronics and exercised in a quiet, distraction-free room with only research personnel present for supervision. Research personnel present in the room did not interact with the participant during treadmill exercise.

Relative VO_2_ (ml kg^-1^ min^-1^), metabolic equivalents (METs), and total kilocalories across all four exercise conditions were estimated by using the American College of Sports Medicine (ACSM) metabolic equation calculation (walking equation: VO_2_ (ml kg^-1^ min^-1^) = 0.1(speed) + 1.8 (speed) (fractional grade) + 3.5 (ml kg^-1^ min^-1^) [38]. Metabolic equivalents were calculated by dividing estimated relative VO_2_ by 3.5 ml kg^-1^ min^-1^ (i.e., one MET), once estimated relative VO_2_ was calculated from the metabolic equation. Estimated relative VO_2_ (ml kg^-1^ min^-1^) was converted to absolute VO_2_ (L min^-1^) and then the following equation was utilized to calculate kilocalories expended: kilocalories minute^-1^ = VO_2_ (L min^-1^) * 5 (Table 2) [38].

**Table 2:** Average METs, VO_2_ (mL kg^-1^ min^-1^), total kilocalories, and pounds of fat across all conditions.

	Control	Texting	Talking	Music
METs	3.3	3.1	3.1	3.6
VO_2_ (mL kg^-1^ min^-1^)	11.5	11.0	11.0	12.4
Total kcal	116	110	110	125

All data are means

### Analytic plan

All data were analyzed with SPSS version 17.0 (SPSS Incorporated, Chicago, IL) with an a-priori α level of ≤ 0.05. Males and females physical characteristics (height, weight, age) were compared using independent samples-t-tests. Because there were no differences between males and females and there were no hypotheses based upon sex, it was not included as an independent variable in all subsequent analysis of variance (ANOVA) models. Four condition (*texting*, *talking*, *music*, *control*) repeated-measures ANOVAs were utilized to examine differences in average treadmill speed, heart rate and liking. Post-hoc analyses for all significant main effects were completed using t-tests with the Benjamini and Hochberg False Discovery Rate correction [39]. NOTE: Differences in estimated relative VO_2_, METs, and kilocalories across all four exercise conditions were not analyzed as each of these variables was ultimately calculated from average treadmill speed. Therefore, analyzing relative VO_2_, METs, and kilocalories using the above ANOVA model would yield the exact same results as the analysis of average treadmill speed. The values calculated from the metabolic calculations (i.e., relative VO_2_, METs, kilocalories) from each exercise condition (*texting*, *talking*, *music*, *control*) are presented in Table 2.

## Results

### Physical characteristics

Independent samples-t-tests revealed no significant differences in males and females physical characteristics for height, weight, and age (Table 1).

### Treadmill speed

There was a significant (*p* < 0.001, effect size (eta-squared (*η* ^2^)) = 0.20) main effect of condition for treadmill speed. Average treadmill speed was significantly greater (*p* ≤ 0.008) in the *music* condition (3.4 ± 1.3 miles hour^-1^) than all other conditions. Average speed during the *control* condition (3.1 ± 1.2 miles hour^-1^) was significantly (*p* ≤ 0.04) greater than the *texting* (2.8 ± 1.1 miles hour^-1^) and *talking* (2.8 ± 1.1 miles hour^-1^) conditions. Average speeds in the *texting* and *talking* conditions were not significantly (*p* = 0.9) different.

### Heart rate

There was a significant (*p* < 0.001, *η* ^2^ = 0.18) main effect of condition for heart rate. Average heart rate was significantly greater (*p* ≤ 0.014) in the *music* condition (122.3 ± 24.3 beats min^-1^) than all other conditions. Average heart rate in the *control* condition (115.4 ± 22.8 beats min^-1^) was significantly (*p* = 0.04) greater than the *texting* condition (109.9 ± 16.4 beats min^-1^) but not (*p* = 0.28) the *talking* condition (112.6 ± 16.1 beats min^-1^). There was a trend (*p* = 0.07) towards a difference in heart rate between the *texting* and *talking* conditions.

### Liking

There was a significant (*p* < 0.001, *η* ^2^ = 0.35) main effect of condition for liking. Average liking was significantly greater (*p* < 0.001) in the *music* condition (7.5 ± 1.5 cm) than all other conditions. Average liking in the *talking* condition (5.4 ± 2.2 cm) was significantly (*p* = 0.05) greater than the *control* condition (4.3 ± 2.2 cm). Liking in the *texting* condition (5.1 ± 2.2 cm) was not different (*p* ≥ 0.10) than the *control* or *talking* conditions.

## Discussion

This study utilized a within-subjects design to compare the intensity (average speed and heart rate) and liking of 30-minute bouts of treadmill exercise during four conditions: texting on a cell phone (*texting*), talking on a cell phone (*talking*), using a cell phone to listen to music (*music*), and no cell phone use (*control*). This design allowed us to test several effects that common cell phone functions may have on a bout of planned exercise. In our previous research, we identified a negative relationship between cell phone use and cardiorespiratory fitness [22]. Interview data collected during that same study suggested a cell phone distraction effect during exercise may partially explain the negative relationship. Present results demonstrated that average treadmill speed and heart rate was significantly greater in the *music* condition compared to all other conditions. This was an expected outcome as previous research has indicated that listening to music may increase exercise intensity [29–33]. Interestingly, average speed in both the *talking* and *texting* conditions was significantly less than the *control* condition. In addition, average heart rate was significantly lower in the *texting* condition relative to the *control*. There was no significant difference between heart rate in the *talking* and *control* conditions. These findings suggests that commonly used cell phone functions (*texting*, *talking*) have the ability to decrease exercise workload (i.e., average treadmill speed), which could negatively impact fitness.

Conversely, two of the cell phone functions tested (*talking* and *music*) increased the liking of a bout of treadmill exercise. Results from this study demonstrated that average liking in the *music* condition was significantly greater than all other conditions. Liking in the *talking* condition was significantly greater than the *control* condition, while liking in the *texting* condition was not significantly different than the *talking* or *control* conditions. Thus, using a cell phone only for *music* increased both intensity (average speed and heart rate) and liking of a bout of treadmill exercise. *Talking* on a cell phone increased the liking of treadmill exercise but reduced walking speed. *Texting*, relative to the *control* condition, reduced speed and heart rate and did not increase liking. This reduction in speed was hypothesized and supports previous findings that used motion capture cameras to assess ground walking (i.e., not on a treadmill) mechanics and speed during cell phone use [15]. In this previous study, the adverse impact of cell phone use on gait mechanics was exacerbated when the task became more difficult, which helps explain why treadmill speed was reduced in both the *talking* and *texting* conditions in the present study. The present findings are also in-line with earlier studies which have identified a distraction effect from cell phone talking and texting which negatively impacts well practiced pedestrian behaviors such as crossing the street [7–14]. Taken together, there appear to be positives and negatives to cell phone use during planned exercise. Because liking has been positively correlated with duration and frequency of exercise as well as long-term commitment towards physical activity [40], [41], results suggest that using the cell phone strictly for listening to *musi*c, and to a lesser degree *talking*, may have benefits on the duration and/or frequency of exercise behavior. Conversely, a lack of time is the most commonly identified barrier to exercise participation in modern society [42–44]. As a result, exercise often occurs in planned bouts of a fixed duration (e.g., 30-minutes). If the duration of a bout of exercise was fixed, using a cell phone for texting and talking during exercise may reduce workload (i.e., speed) without the possibility of increasing duration. This may provide a partial explanation for the negative link between cell phone use and cardiorespiratory fitness that our group has previously identified.

In addition to the potential implications cell phone use during exercise may have on intensity and ultimately cardiorespiratory fitness, these altered exercise intensities may have important implications for caloric balance. Many individuals utilize exercise as a means of preventing weight gain [45]. Based upon average treadmill speed, caloric expenditure was greater in the *music* condition (125 kcals 30 min^-1^) compared to all other conditions in the present study. Caloric expenditure in both the *texting* and *talking* conditions were the same (110 kcals 30 min^-1^ each condition), which was significantly less than the *control* condition (116 kcals 30 min^-1^). Assuming an individual participates in the American College of Sports Medicine’s recommended 150 min week^-1^ of moderate intensity exercise for an entire year and that 3,500 kcal = one pound bodyweight [38], the energy expenditure from each condition for a year would be equivalent to 9.3 lb for the *music* condition, 8.6 lb for the *control* condition and 8.2 lb for the *talking* and *texting* conditions. In other words, using a cell phone to listen to music while exercising may result in 0.7 lb or 1.1 lb greater energy expenditure per year than not using a cell phone or *talking*/*texting*, respectively. Not using a phone at all during exercise may result in 0.4 lb greater energy expenditure per year than *talking*/*texting*. These differences may seem small however, weight gain for most individuals is a slow process. American adults gain an average one pound of body weight per year [45]. Over time this slow weight gain can lead to the development of obesity. Therefore, making simple, small changes such as using your cell phone to listen to music and/or avoiding texting or talking on the phone during exercise may help increase exercise intensity and caloric expenditure which could combat this slow weight gain.

One important caveat to these potential differences in caloric expenditure is the fact that despite a significantly slower average speed in the *talking* condition participants exhibited a heart rate that was not lower from the *control* condition. This is somewhat surprising as we would expect a reduced heart rate to accompany a reduced workload (i.e., lower speed) as heart rate is a known correlate to exercise intensity [46]. This was the case in the *texting* condition as participants exhibited both a significantly slower speed and lower average heart rate relative to the *control* condition. It may be that that the energy requirements for talking and holding the phone to their ear (speaker-phone mode was not allowed) further increased participants’ heart rate making the overall intensity of the *talking* condition similar to the *control* condition despite a reduced average speed. If this was the case, the metabolic cost of the *control* and *talking* conditions may not be dissimilar despite the differences in average treadmill speed.

While we believe this study yields novel and useful information, it is not without limitations. Presently we only examined treadmill exercise, which may not be the exercise mode of choice for all participants, thereby limiting our ability to generalize the current results to other modes of exercise. Requiring participants to exercise on a treadmill may have reduced intensity and liking if this was not the preferred mode of exercise. Additionally, we utilized interviews during the *talking* and *texting* conditions that asked questions which were unrelated to the activity the participants were undertaking: exercise. Recent research has indicated that if a cell phone user is participating in an action while interacting with another individual via cell phone that interaction may be less distracting if both cell phone users are aware of the action being participated in [47]. For example, if a coach/trainer were to interact with an athlete via cell phone during exercise and that interaction is relevant to the exercise (e.g., providing instruction/encouragement) that interaction may not distract the athlete from their task. This type of cell phone interaction may have a different effect on exercise behavior than was noted in the present study and is an idea that warrants further research. Another potential limitation of the present study may be related to the *music* condition. While listening to a self-selected playlist of music likely best mimics a real-world exercise environment, we did not attempt to assess the type (genre, tempo, loudness, etc.) of the music participants listened to. This may have been useful data as music tempo and loudness have been shown to have an effect on exercise intensity [34–36]. The sample also consisted of only college students which have been raised entirely in the digital age, while other populations (middle-aged and older adults) may have differing results due to the possibility of having less experience and comfort associated with digital technology, thus limiting our ability to generalize these results to other populations. That said, previous research has measured a significant cell phone distraction effect in early adolescents [11], college students [10], and older adults [7]. Of the various populations studied, the negative impacts appear greatest with older adults as the cognitive decline associated with aging may exacerbate the dual-task effect [7]. Lastly, we estimated caloric expenditure from treadmill speed. This may underestimate actual energy expenditure in the *talking* condition as it does not account for the participants’ caloric cost of talking and holding the phone to their ear. Future studies may wish to utilize other methods (e.g., oxygen consumption via indirect calorimetry) to obtain a more accurate caloric cost of the various conditions.

## Conclusions

Cell phones are ubiquitous among today’s young adults [48], [49]. However, we are only just now beginning to discover some of the behavioral effects that the use of these devices may have. Because modern cell phones allow individuals to access activities (e.g., browsing the internet, playing video games, watching videos, etc.) that have traditionally been negatively associated with physical activity behavior it is possible that the use of these devices may distract from or interfere with physical activity behavior and planned exercise. Presently, we have demonstrated that using a cell phone for *talking* or *texting* during treadmill exercise significantly reduces the workload (i.e., average speed) of that exercise relative to a condition where no cell phone was used. Conversely, using a cell phone to listen to a self-selected playlist of *music* significantly increased exercise intensity (both speed and heart rate) relative to all other conditions. Relative to the *control* condition, both listening to *music* and *talking* on the phone increased liking of the exercise bout which may have positive effects on exercise duration and/or frequency assuming the individual has the flexibility in their daily lives to exercise for a longer duration and/or more frequently. In conclusion, relative to a *control* condition different cell phone functions appear to differentially affect exercise behavior. Listening to *music* increased the intensity and liking of the exercise bout. *Talking* increased liking, maintained heart rate but reduced workload. *Texting* reduced intensity (both speed and heart rate) and did not alter liking. Therefore, the relationship between cell phone use and exercise intensity appears to be specific to the function being utilized on the phone. Individuals should take note of these differences when utilizing their cell phones during their planned exercise time.

## References

[1] Harrison MA, Gilmore AL. U txt WHEN? College students’ social contexts of text messaging. Soc Sci. 2012;49(4): 513–518.

[2] Tindell DR, Bohlander RW. The use and abuse of cell phones and text messaging in the classroom: A survey of college students. Coll Teaching. 2011;60(1): 1–9.

[3] Hancock PA, Lesch M, Simmons L. The distraction effects of phone use during crucial driving maneuver. Accident Anal Prev. 2003;35(4): 501–514. 12729814 10.1016/s0001-4575(02)00028-3

[4] Horrey WJ, Wickens CD. Examining the impact of cell phone conversations on driving using meta-analytic techniques. Hum Factor Ergon Soc. 2006;48(1): 196–205. 16696268 10.1518/001872006776412135

[5] Rakauskas ME, Gugerty LJ, Ward NJ. Effects of naturalistic cell phone conversations on driving performance. J Safety Res. 2004;35(4): 453–464. 15474548 10.1016/j.jsr.2004.06.003

[6] Strayer DL, Drews FA, Crouch DJ. A comparison of the cell phone driver and the drunk driver. Hum Factor Ergon Soc. 2006;48(2): 381–391. 10.1518/001872006777724471 16884056

[7] Neider MB, Gaspar JG, McCarley JS, Crowell JA, Kaczmarski H, Kramer AF. Walking and talking: Dual-task effects on street crossing behavior in older adults. Psychol Aging. 2011;26(2): 260–268. 10.1037/a0021566 21401262 PMC3699858

[8] Neider MB, McCarley JS, Crowell JA, Kaczmarski H, Kramer AF. Pedestrians, vehicles, and cell phones. Accident Anal Prev. 2010;42: 589–594. 10.1016/j.aap.2009.10.004 20159083

[9] Schwebel DC, Stavrinos D, Byington KW, Davis T, O’Neal EE, de Jong D. Distraction and pedestrian safety: How talking on the phone, texting, and listening to music impact crossing the street. Accident Anal Prev. 2012;45: 266–271. 10.1016/j.aap.2011.07.011 22269509 PMC3266515

[10] Stavrions D, Byington KW, Schwebel DC. Distracted walking: Cell phones increase injury for college pedestrians. J Saf Res. 2011;42: 101–107. 10.1016/j.jsr.2011.01.004 21569892

[11] Stavrinos D, Byington KW, Schwebel DC. Effect of cell phone distraction on pediatric pedestrian injury risk. Pediatr. 2010;209: e179–e185. 10.1542/peds.2008-1382 19171568

[12] Hyman IE Jr, Boss MS, Wise BM, McKenzie KE, Caggiano JM. Did you see the unicycling clown? Inattentional blindness while walking and talking on a cell phone. Appl Cognit Psychol. 2010;24: 597–607.

[13] Thompson LL, Rivara FP, Ayyagari RC, Ebel BE. Impact of social and technological distraction on pedestrian crossing behaviour: An observational study. Inj Prev. 2013;19: 232–237. 10.1136/injuryprev-2012-040601 23243104 PMC3717764

[14] Nasar J, Hecht P, Wener R. Mobile telephones, distracted attention, and pedestrian safety. Accident Anal Prev. 2008;40: 69–75. 10.1016/j.aap.2007.04.005 18215534

[15] Parr ND, Hass CJ, Tillman MD. Cellular phone texting impairs gait in able-bodied young adults. J Appl Biomech. 2014;30: 685–688. 10.1123/jab.2014-0017 25010143

[16] Nasar JL, Troyer D. Pedestrian injuries due to mobile phone use in public places. Accident anal Prev. 2013;57: 91–95. 10.1016/j.aap.2013.03.021 23644536

[17] Junco R, Cotton SR. No A 4 U: The relationship between multitasking and academic performance. Comput Edu. 2012;59: 505–514.

[18] Jacobsen WC, Forste R. The wired generation: Academic and social outcomes of electronic media use among university students. Cyberpsychology, Behav, Soc Networking. 2011;14(5): 275–280. 10.1089/cyber.2010.0135 20961220

[19] Sanchez-Martinez M, Otero A. Factors associated with cell phone use in adolescents in the community of Madrid (Spain). Cyberpsychology, Behav, Soc Networking. 2009;12: 131–137. 10.1089/cpb.2008.0164 19072078

[20] Lepp A, Barkley J, Karpinski A. The relationship between cell phone use, academic performance, anxiety, and satisfaction with life in college students. Comput Hum Behav. 2014;31: 343–350.

[21] Dietz S, Henrich C. Texting as a distraction to learning in college students. Comput Hum Behav. 2014;36: 163–167.

[22] Lepp A, Barkley JE, Sanders GJ, Rebold M, Gates P. The relationship between cell phone use, physical activity behavior, and cardiorespiratory fitness in a sample of U.S. college students. Int J Behav Nutr Phys Act. 2013;10: 79 10.1186/1479-5868-10-79 23800133 PMC3693866

[23] Must A, Tybor DJ. Physical activity and sedentary behavior: A review of longitudinal studies of weight and adiposity in youth. Int J Obes Relat Metab Disord. 2005;29(S2): S84–S96. 10.1038/sj.ijo.0803064 16385758

[24] Ford E, Kohl H III, Mokdad A, Ajani UA. Sedentary behavior, physical activity, and the metabolic syndrome among U.S. adults. Obes Res. 2005;13: 608–614. 15833947 10.1038/oby.2005.65

[25] Tremblay MS, LeBlanc AG, Kho ME, Saunders TJ, Larouche R, Colley RC, et al Systematic review of sedentary behaviour and health indicators in school-aged children and youth. Int J Behav Nutr Phys Act. 2011;8: 98 10.1186/1479-5868-8-98 21936895 PMC3186735

[26] Santos R, Mota J, Okley AD, Pratt M, Moreira C, Coelho-E-Silva MJ, et al. The independent associations of sedentary behaviour and physical activity on cardiorespiratory fitness. BR J Sports Med. 2013; In press. 10.1136/bjsports-2012-091610 23410883

[27] Myers J, Prakash M, Froelicher V, Do D, Partington S, Atwood JE. Exercise capacity and mortality among men referred for exercise testing. N Engl J Med. 2002;346: 793–801. 11893790 10.1056/NEJMoa011858

[28] Carnethon MR, Gidding SS, Nehgme R, Sidney S, Jacobs DR Jr, Liu K. Cardiorespiratory fitness in young adulthood and the development of cardiovascular disease risk factors. JAMA. 2003;290: 3092–3100. 14679272 10.1001/jama.290.23.3092

[29] Foster C, Porcari J. Exploring the effects of music on exercise intensity. American Council on Exercise. 2010; Available: https://www.acefitness.org/certifiednews/images/article/pdfs/MusicStudy.pdf. Accessed 3 March 2014.

[30] Hagen J, Foster C, Rodriguez-Marroyo J, de Koning JJ, Mikat RP, Hendrix CR, et al The effect of music on 10-km cycle time-trial performance. Int J Sports Physiol Perform. 2013;8: 104–106. 22868289 10.1123/ijspp.8.1.104

[31] Karageorghis CI, Mouzourides DA, Priest DL, Sasso TA, Morrish DJ, Walley CJ. Psychophysical and ergogenic effects of synchronous music during treadmill walking. J Sport Exerc Psychol. 2009;31: 18–36. 19325186 10.1123/jsep.31.1.18

[32] Mohammadzadeh H, Tartibiyan B, Ahmadi A. The effects of music on perceived exertion rate and performance of trained and untrained individuals during progressive exercise. J Phys Educ Spor. 2008;6: 67–74.

[33] Priest DL, Karageorghis CI, Sharp NCC. The characteristics and effects of motivational music in exercise settings: the possible influence of gender, age, frequency of attendance, and time of attendance. J Sports Med Phys Fitness. 2004;44: 77–86. 15181394

[34] Edworthy J, Waring H. The effects of music tempo and loudness on treadmill exercise. Ergon. 2006;49(15): 1597–1610. 17090506 10.1080/00140130600899104

[35] Ruscello B, D’Ottavio S, Padua E. The influence of music on exercise in a group of sedentary elderly women: An important tool to help the elderly to stay active. J Sports Med Phys Fitness. 2014;54(4): 536–544. 25034556

[36] Leman M, Moelants D, Varewyck M, Styns F, van Noorden L, Martens JP. Activating and relaxing music entrains the speed of beat synchronized walking. PLOS ONE. 2013;8(7): e67932 10.1371/journal.pone.0067932 23874469 PMC3707869

[37] Terry PC, Karageorghis CI, Saha AM, D’Auria S. Effects of synchronous music on treadmill running among elite triathletes. J Sci Med Sport. 2012;15(1): 52–57. 10.1016/j.jsams.2011.06.003 21803652

[38] Whaley MH, Brubaker PH, Otto RM. American College of Sports Medicine ACSM’s Guidelines for Exercise Testing and Prescription (7th ed.). Philadelphia, PA: Lippincott, Williams, and Wilkins; 2006.

[39] Benjamini Y, Hochberg Y. Controlling the false discovery rate: a practical and powerful approach to multiple testing. J R Stat Soc Series B. 1995;57(1): 289–300.

[40] Hagberg LA, Lindahl B, Nyberg L. Importance of enjoyment when promoting physical exercise. Scand J Med Sci Spor. 2009;19(5): 740–747. 10.1111/j.1600-0838.2008.00844.x 18694433

[41] Salmon J, Owen N, Crawford D, Bauman A, Sallis JF. Physical activity and sedentary behavior: a population-based study of barriers, enjoyment, and preference. Health Psychol. 2003;22: 178–188. 12683738 10.1037//0278-6133.22.2.178

[42] Disman RK, Sallis JF, Orenstein DR. The determinants of physical activity and exercise. Public Health Rep. 1985;100(2): 158–171. 3920714 PMC1424729

[43] King AC, Taylor CB, Haskell WL, DeBusk RF. Identifying strategies for increasing employee physical activity levels: Findings from the Stanford/Lockheed Exercise Survey. Health Educ. Q. 1990;17(3): 269–285. 2228630 10.1177/109019819001700304

[44] Sherwood NE, Jeffery RW. The behavioral determinants of exercise: Implications for physical activity interventions. Annu Rev Nutr. 2000;20: 21–44. 10940325 10.1146/annurev.nutr.20.1.21

[45] Markland D, Hardy L. The exercise motivations inventory: preliminary development and validity of a measure of individuals’ reasons for participation in regular physical exercise. Pers Indiv Differ. 1993;15(3): 289–296.

[46] Karvonen J, Vuorimaa T. Heart rate and exercise intensity during sports activities. Sports Med. 1988;5(5): 303–311. 3387734 10.2165/00007256-198805050-00002

[47] Gaspar JG, Street WN, Windsor MB. Providing views of the driving scene to drivers’ conversation partners mitigates cell-phone-related distraction. Psychol Sci. 2014;25(12): 2136–2146. 10.1177/0956797614549774 25304886

[48] Palfrey J, Gasser U. Born Digital: Understanding the First Generation of Digital Natives. New York (NY): Basic Books; 2008.

[49] Anderson J, Rainie L. Millennials will benefit and suffer due to their hyperconnected lives. The Pew Research Center’s Internet and American Life Project. 2012; Available: http://www.pewinternet.org/Reports/2012/Hyperconnected-lives/Overview.aspx. Accessed 2013 Mar 3.

